# Novel golf prosthesis for bilateral upper limb loss: design, fabrication, and biomechanical evaluation

**DOI:** 10.3389/fspor.2026.1766695

**Published:** 2026-05-08

**Authors:** Jason T. Maikos, Leif M. Nelson, David V. Herlihy, Christopher M. Fantini, Jonathan J. Glasberg, John M. Chomack, Alexis N. Sidiropoulos

**Affiliations:** 1 Prosthetics and Sensory Aids Service, Veterans Affairs New York Harbor Healthcare System, New York, NY, United States; 2 National Veterans Sports Programs and Special Events, Department of Veterans Affairs, Washington, DC, United States; 3 Narrows Institute for Biomedical Research and Education, Inc., Brooklyn, NY, United States; 4 Orthotic, Prosthetic & Pedorthic Clinical Services, Department of Veterans Affairs Rehabilitation & Prosthetics Services, Washington, DC, United States

**Keywords:** adaptive sports, bilateral upper limb loss, biomechanics, golf biomechanics, golf prosthesis, sport-specific prosthesis, swing kinematics, upper limb loss

## Abstract

**Introduction:**

Individuals with bilateral upper limb loss face substantial barriers to independence and recreational participation. Adaptive sports can enhance psychological and physical well-being, yet few prosthetic devices are specifically designed or biomechanically evaluated for use in adaptive sports. Golf demands coordinated motion, stability, and dynamic control, often exceeding the capabilities of most traditional prostheses. Custom prosthetic solutions remain essential for enabling meaningful participation. This study aimed to design, fabricate, and evaluate a custom golf prosthesis for a veteran with bilateral upper limb loss, with primary emphasis on functional feasibility and characterization of swing biomechanics.

**Methods:**

A custom carbon-fiber monolimb prosthesis with a hinged clamshell distal end was developed to secure the golf club. Following iterative fittings, biomechanical data were collected during driver swings to quantify body alignment, downswing kinematics, X-factor rotation, and weight shift relative to published normative datasets.

**Results:**

The custom prosthesis enabled independent club attachment, a coordinated two-armed golf swing, and participation in golf activities. Biomechanical analysis demonstrated reduced pelvis, torso, and lead arm angular velocities, diminished X-factor rotation, lower clubhead speed, and altered follow-through loading patterns relative to normative values.

**Discussion:**

The device demonstrated functional feasibility for adaptive golf participation in an individual with bilateral upper limb loss. This single-participant evaluation highlights important biomechanical considerations related to wrist orientation, load transfer, and stabilization in sport-specific prosthetic design. Findings support the role of user-centered, clinically feasible fabrication approaches in expanding access to adaptive sports while informing future biomechanical refinement and longitudinal outcome evaluation.

## Introduction

Individuals with major upper limb loss often face challenges that negatively impact activity, body function, and participation in activities (1). Device abandonment remains common, with one large survey indicating that approximately one in five upper-limb prosthesis users reject their prosthesis (2), often due to dissatisfaction with limited function (3). These challenges are magnified for individuals with bilateral upper limb loss, where the absence of a sound limb complicates daily activities, increases reliance on prosthetic or assistive devices, and limits opportunities for independent participation (4). Compared with unilateral upper limb loss, bilateral upper limb loss has also been associated with greater disability and psychosocial burden (5).

Beyond challenges with daily tasks, individuals with upper limb loss can encounter significant barriers to engaging in physical activities, which are associated with activity restriction and poorer physical and psychosocial outcomes (6). Conversely, participation in sports and physical activity has been linked to improved psychological health, social integration, and musculoskeletal conditioning (7, 8). Adaptive sports can reduce access barriers, improve quality of life, and serve as an effective rehabilitation tool (9–12). However, sustained involvement requires prosthetic solutions that are both functional and user specific. Although several commercially available terminal devices exist to support participation in adaptive sports (13, 14), they are rarely tailored to individual goals, and few have undergone rigorous scientific testing to establish functional efficacy (15). Customized prosthetic solutions therefore remain essential for enabling meaningful sport participation.

Golf is particularly well suited for adaptive play for a wide array of impairment groups, offering both physical (e.g., balance, coordination, and cardiovascular activity) and psychosocial (e.g., social participation and stress reduction) benefits (16). For individuals with upper limb loss, golf typically requires a one-arm swing or a specialty terminal device to securely hold and swing the club (17–19). Effective designs must be lightweight, durable, adjustable for different clubs, and easy to don and doff. Commercially available devices, such as the Eagle and Eagle Flex (TRS Inc., Boulder, CO) and the Helix (Fillauer, Inc., Chattanooga, TN), are designed primarily for use in unilateral limb loss and rely on distal terminal device interfaces to secure the club. This may limit adaptability and stability across different clubs and swing styles, particularly for individuals with bilateral upper limb loss. These commercial devices may not be universally suitable, leaving a critical gap in prosthetic solutions for this population. A small number of non-commercial, custom prosthetic designs have been described in the literature, demonstrating the feasibility of individualized solutions (18, 19); however, these devices often require club modifications, segmented distal attachments, or highly specialized fabrication approaches that may limit reproducibility in typical clinical prosthetic settings (19). To date, there remains a paucity of peer-reviewed reports describing golf-specific prostheses or terminal devices for individuals with bilateral upper limb loss, and no studies have reported a full-body biomechanical evaluation of such devices in this population.

For golfers with bilateral upper limb loss, the absence of a sound limb removes potential compensatory strategies, likely complicating tasks such as donning equipment, stabilizing the club, and coordinating swing mechanics. Athletes in this population require prosthetic solutions that simultaneously restore grip, provide stability, and allow for dynamic movement while remaining practical to fabricate, which are needs not fully met by existing devices. To address this gap, this study developed and biomechanically evaluated a golf-specific prosthesis for a veteran with bilateral upper limb loss that integrates the club directly into a monolimb socket-clamshell structure, enabling control through the residual limb rather than via distal attachment interfaces alone. Mechanistically, this design enables club stabilization through the socket structure itself and avoids club modification, while functionally supporting rapid donning/doffing and consistent alignment across clubs. Biomechanical data were collected using full-body motion capture to characterize swing mechanics and device function. It was hypothesized that the prosthesis would enable the participant to perform a coordinated golf swing and generate measurable swing mechanics while revealing biomechanical differences relative to normative golf swing data. Importantly, this study represents, to our knowledge, the first full-body biomechanical characterization of a golf-specific prosthesis used by an individual with bilateral upper limb loss. The primary emphasis was feasibility and functional participation rather than comparative performance, while also identifying opportunities for future work incorporating objective, longitudinal, and user-reported outcome measures.

## Materials and methods

### Case description

A 67-year-old male veteran with bilateral upper limb loss secondary to trauma during military service in Vietnam presented to the prosthetics clinic at Veterans Affairs New York Harbor Healthcare System (VANYHHS) with a primary goal of golfing recreationally. The participant was 1.8 m tall and weighed 83.9 kg. He presented with a left wrist disarticulation–level residual limb and a short transradial residual limb on the right. His left and right prostheses each consisted of self-suspending sockets with laminated frames and myoelectrically controlled wrist and hand components. He was right-hand dominant prior to limb loss; following bilateral upper limb loss, functional dominance was trained on the left side due to longer residual limb length. He described himself as a recreational, inexperienced golfer with no formal training and limited recent playing history. Analysis of this single-participant study was approved by the VANYHHS Institutional Review Board (protocol ID 1628), which also granted a waiver of documentation of informed consent due to the retrospective nature of the analysis.

**Figure 1:**
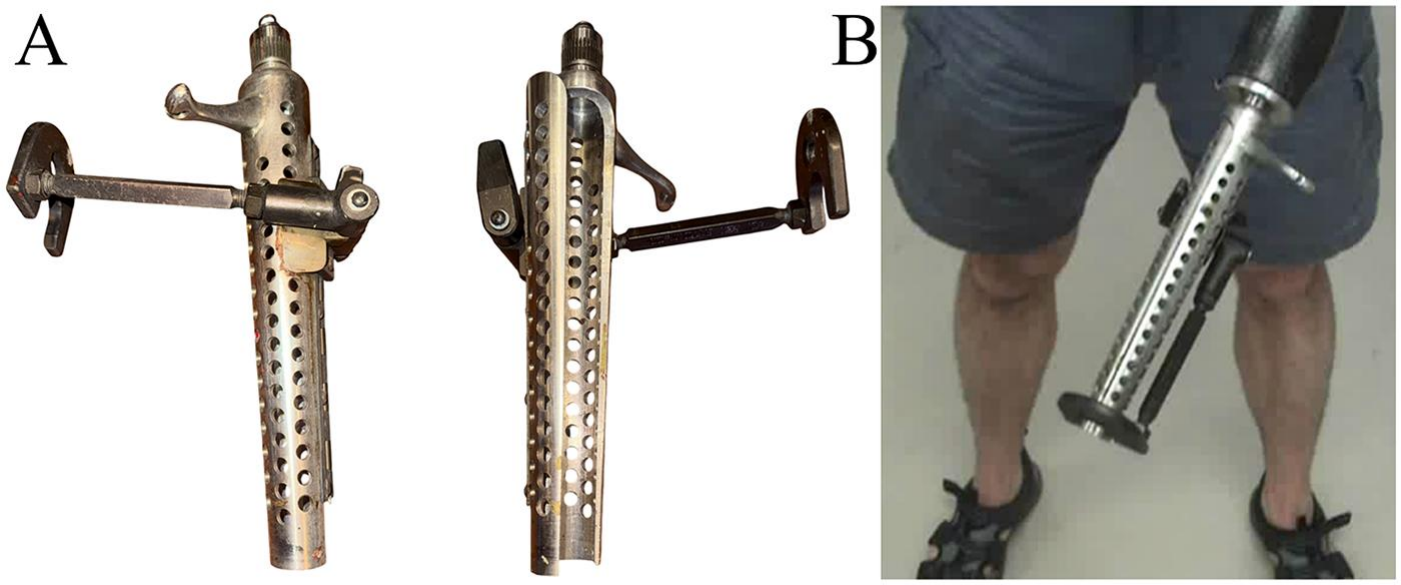
Custom-fabricated steel terminal device for adaptive golfing, originally fabricated in the 1970s. **(A)** Device shown in the open and closed positions. **(B)** Device attached to the participant's prosthetic socket.

**Figure 2:**
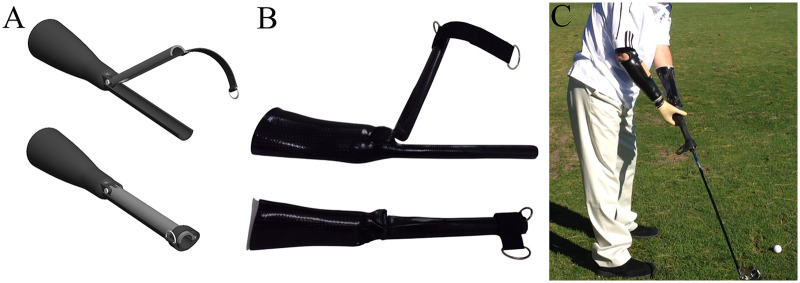
Design and development of the custom golf prosthesis. **(A)** Three-dimensional digital model of the custom monolimb prosthesis with the clamshell distal end shown in the open and closed positions; the distal end is secured using a Dacron strap and D-ring. **(B)** Fabricated carbon-fiber monolimb prosthesis with hinged clamshell distal end in the open and closed positions. **(C)** Participant demonstrating prosthesis use during the backswing on the golf course. The definitive prosthesis included a flexible inner socket with suction suspension, carbon-fiber and fiberglass lamination, and a Dacron strap closure.

At presentation, the participant previously used a custom-fabricated steel terminal device for recreational golfing, which was fabricated in the 1970s (Figure 1) and had not been used for over 30 years. It measured approximately ten inches in length and attached to the left prosthesis wrist unit via a commercially available quick disconnect collar. Its heavy weight caused discomfort in the left residual limb, and the internal diameter was too narrow to accommodate the participant's current golf clubs. The terminal device opened laterally via a hinge on the opposite side of the cylinder from the opening. Following consultation, a new lightweight golf prosthesis was prescribed to enable golf participation. The approach was to design a one-piece, lightweight carbon fiber prosthesis for the left residual limb with a custom, clamshell distal end to allow easy attachment to the golf clubs (Figure 2). Due to its one-piece construction, this design aimed to withstand the forces and torques associated with a typical golf swing, specifically during impact.

### Golf prosthesis design

The design featured a monolimb outer frame with a customized clamshell distal end that fully encompassed the golf club grip (Figure 2A). The inferior portion of the prosthesis was a direct, fixed extension of the socket frame, while the superior portion of the clamshell hinged to open and close around the club. The internal aspect of the clamshell was dimensioned from a direct cast of the participant's golf club (driver), which was a standard-length, commercially available club (Tommy Armour Silver Scot Series) appropriate for his stature. The shaft grip sizes were also consistent across his set. A distally positioned polyester webbing strap (Dacron) with hook-and-loop fastener (Velcro) and D-ring allowed the participant to secure and release the club independently using his right prosthesis. Clubs were press-fit into the clamshell, generating friction to prevent rotation during swings. This design required no modification to the golf clubs, making the customized distal end universal across the entire set of clubs. No wrist unit was incorporated, resulting in a fixed, neutral wrist orientation.

A perpendicular handle was initially incorporated at the mid-socket level to allow stabilization of the golf prosthesis by the right prosthetic hand during the swing. After preliminary testing, the participant preferred to remove the handle and stabilize by directly gripping the golf prosthesis with the right myoelectric hand palm-up, in approximately 180° of rotation. Prior to fabrication, consideration was given to the angular position of the customized distal end with respect to the residual limb. A 0^o^ angle was used in the prosthetic design to meet the participant's needs during initial fabrication.

Design constraints and trade-offs were intentionally balanced to prioritize safe, stable swing execution and clinical feasibility. A monolimb architecture was selected to provide a continuous structural frame from socket to distal interface, improving load tolerance at impact, simplifying fabrication, and eliminating the need for club modification or length compensation. This approach also supported rapid interchangeability across standard clubs while maintaining consistent alignment. The fixed wrist orientation and absence of a wrist unit further improved structural rigidity, socket comfort during high-torque loading at impact, and durability while reducing distal mass and fabrication complexity. However, this configuration was expected to limit wrist-mediated contributions to clubhead acceleration and rotational sequencing, representing a trade-off between distal mobility and proximal stability. Together, these considerations highlight the design balance required when developing sport-specific prostheses for high-load, ballistic movements.

### Golf prosthesis fabrication

The left residual limb was measured and cast in neutral pronation-supination. A plaster mold was created from the cast, and a diagnostic socket (Vivak plastic) was fabricated. Following initial fitting, a second diagnostic socket was fabricated to reduce overall volume for fit and comfort, which was confirmed after fitting. The definitive prosthesis was fabricated using a flexible thermoplastic material, light weight carbon fiber (3 layers), fiberglass (3 layers), epoxy/acrylic resin, heavy duty cabinet hinges, a polyester webbing strap, D-ring, and hook-and-loop fastener (Figure 2B). A flexible inner socket incorporated a one-way air expulsion valve for suction suspension. Socket trim lines were below the epicondyles, with secondary suspension over the residual anatomical wrist styloids.

### Prototype testing

Following fabrication, the prosthesis was tested at the Veterans Integrated Service Network (VISN) 2 Biomechanics Research for the Advancement of Veteran Outcomes (BRAVO) Laboratory at VANYHHS. This preliminary testing was conducted as part of the iterative design and adaptation process to confirm fit, comfort, and functional operation prior to formal biomechanical testing. The participant donned the prosthesis and secured his driver (Tommy Armour Silver Scot Series) in the clamshell distal end (Figure 2C). The right prosthesis, equipped with a self-suspending laminated socket, electric wrist rotator (10S17, Ottobock, Duderstadt, Germany) and SensorHand Speed (Ottobock, Duderstadt, Germany), was used to grip the left prosthetic socket for stabilization during the swing. During initial testing, the participant performed 20 golf swings from an artificial turf mat, striking golf balls into a practice net. Observations included mild right residual limb irritation, which prompted socket adjustments (cutouts at the olecranon and epicondyles) to reduce torsional forces in these areas. A Motion Control Multi-Flex Wrist (Fillauer, Inc., Chattanooga, TN, USA) was incorporated into the right prosthesis to allow passive wrist flexion and extension and ulnar and radial deviation with powered wrist rotation for enhanced gripping of the golf prosthesis. These adaptations ensured that the prosthesis could be used safely and functionally, enabling a coordinated two-armed golf swing, independent club manipulation, and participant comfort during preliminary testing.

### Data collection

The participant returned to the VISN 2 BRAVO Laboratory as part of an adaptive sports clinic (20), which included instructional and on-course golf activities. Biomechanical data were collected during the clinic in a controlled laboratory setting using an 8-camera motion capture system (Vicon Inc., Oxford, UK) at 120 Hz and two force platforms (AMTI, Waterford, MA, USA) at 1,200 Hz. A six-degree-of-freedom marker set was placed on the participant as previously described (20), with the radial and ulnar styloid markers relocated to the proximal base of the clamshell component to approximate the wrist styloid locations. Because the monolimb prosthesis functioned as a rigid body with no wrist articulation, small shifts in marker location were not expected to affect joint angle or angular velocity calculations, as the segment moved as a single rigid unit regardless of marker location. Wrist kinematics remained neutral throughout the swing independent of marker placement, and angular velocities of the distal segment were preserved under rigid-body assumptions. Eight markers were affixed to the golf club (five on the shaft, one at the shaft-head interface, and two on the clubhead) and the golf ball was covered in reflective tape. A single session of data was collected. After five practice swings for acclimation, the participant performed six recorded swings using the driver off an artificial turf mat, while standing in his natural stance with one foot on each force plate.

### Data processing

**Figure 3:**
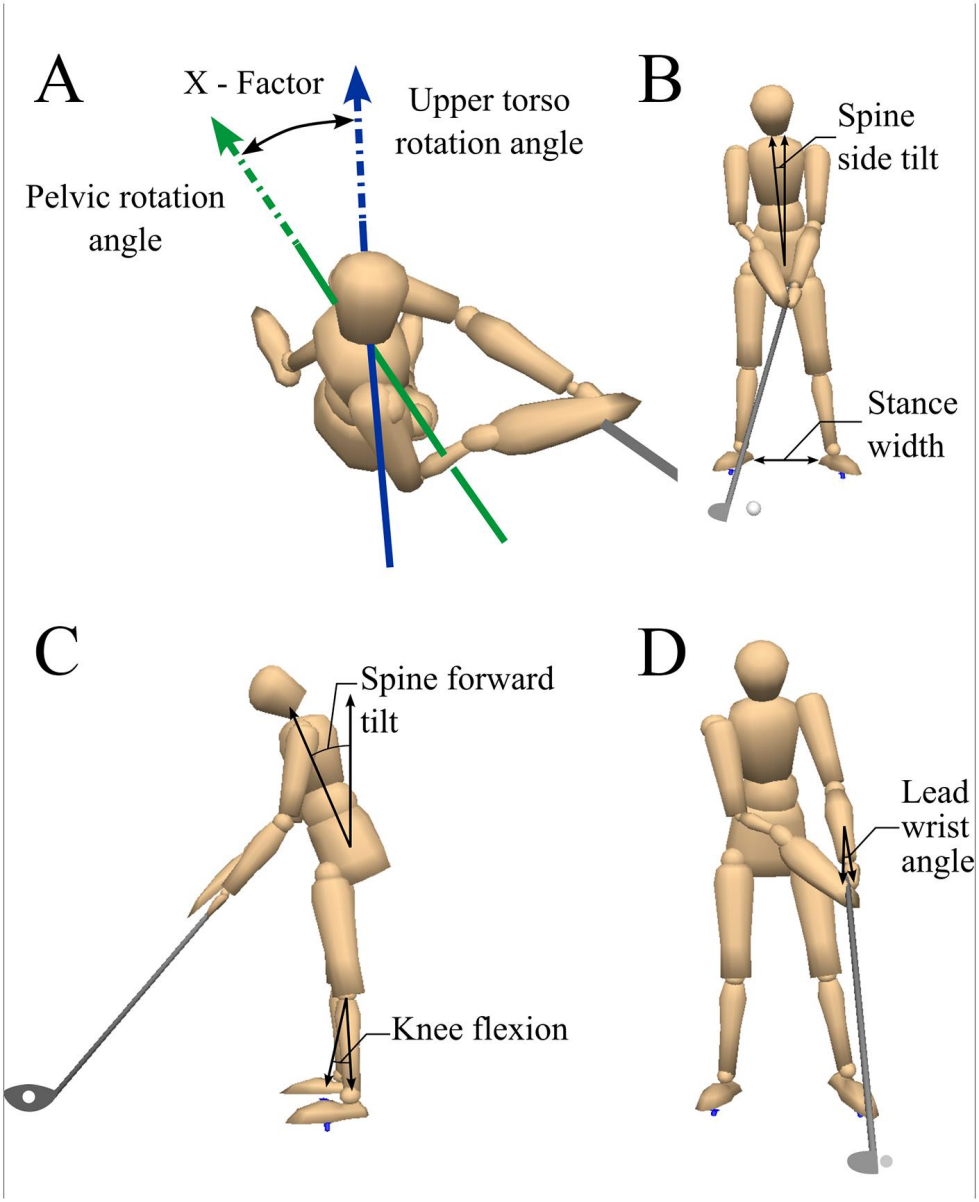
Biomechanical parameters extracted during golf swings. **(A)** Pelvis-thorax separation angle (X-factor). **(B)** Spine side tilt angle and stance width at address. **(C)** Spine forward tilt and knee flexion at address. **(D)** Lead wrist angle at address. Illustrations depict orientation definitions used for quantitative analysis.

Biomechanical data were collected with Vicon Workstation software (Vicon, Oxford, UK) and processed using Visual3D (C-Motion, Germantown, MD). The golf swing was segmented into four phases: address (stance before club movement), transition (the point of change from backswing to downswing), impact (clubhead-ball contact), and follow-through (post-impact to swing completion) (20). Extracted biomechanical parameters (Figure 3) included body position at address, downswing kinematics, pelvis-thorax X-factor (defined as the angle between the line through the right and left anterior superior iliac spines and the line through the right and left acromion processes in the axial plane), and weight shift between the lower limbs. Definitions and orientations for each parameter are summarized in Supplementary Table S1.

### Data analysis

Given the single-participant design, no inferential statistical analyses were performed; all results are descriptive observations specific to this individual. Comparisons made to published normative data provide context for observed deviations. These comparisons are intended as illustrative reference points, not as direct measures of device efficacy. Normative datasets were selected because they provide the most comprehensive published benchmarks for the parameters of interest. Comparable recreational or age-matched datasets were not available across all variables examined.

## Results

A summary of all measured biomechanical variables, including participant values and corresponding normative ranges, is provided in Table 1. Key findings for body alignment, downswing kinematics, X-factor rotation, and weight-shift patterns are described below.

**Table 1:** Biomechanical parameters during golf address and swing compared with normative values.

Body alignment			
Body position at address		Measured values, mean (SD)	Normative values
Stance width (cm)		46.5 (0.8)	51.8^a^
Stance percentage of shoulders (%)		139.3 (4.7)	100^b^
Spine forward tilt (deg)		3.3 (0.8)	28^c^
Spine side tilt (deg)		4.0 (1.3)	6^c^
Lead knee flexion (deg)		3.0 (4.1)	20−25^d^
Trail knee flexion (deg)		2.5 (3.4)	20−25^d^
Downswing parameters			
Parameter		Measured values, mean (SD)	Normative values
Maximum pelvis angular velocity (deg/s)		91.5 (13.9)	280−590^e,f,g^
Maximum torso angular velocity (deg/s)		30.5 (6.9)	470−760^e,f,g^
Maximum lead arm angular velocity (deg/s)		26.3 (17.9)	1000−1700^g^
Maximum club angular velocity (deg/s)		156.5 (50.1)	2,000−2,413^h^
Lead wrist angle at impact (deg)		2.7 (2.9)	13.9–35^d,i^
Maximum club speed (m/s)		21.5 (1.9)	36−52^g,j^
X-factor			
Segment		Measured values, Mean (SD)	Normative values
Pelvis (deg)		−26.7 (3.8)	−37 to −51^k,l^
Thorax (deg)		−27.2 (4.0)	−39 to −52^k,l^
Weight Shift			
Phase	Limb	Measured values, %, (SD)	Normative values, %
Address	Lead	50.8 (2.2)	50^k^
	Trail	49.2 (2.2)	50^k^
Transition	Lead	16.0 (2.4)	20^k^
	Trail	84.0 (2.4)	80^k^
Impact	Lead	60.5 (8.6)	80^k^
	Trail	39.5 (8.6)	20^k^
Follow-through	Lead	41.5 (5.9)	70^k^
	Trail	58.5 (5.9)	20^k^

Measured values are reported as mean (SD). Normative ranges are drawn from previously published golf biomechanics literature: ^a^(42), ^b^(43), ^c^(21), ^d^(44), ^e^(22), ^f^(23), ^g^(24), ^h^(45), ^i^(46), ^j^(25), ^k^(26), ^l^(47).

### Body alignment at address

At address, the participant adopted a more upright posture relative to normative values in skilled golfers. Spine forward tilt was markedly reduced at 3.3° (0.8°) relative to typical values of approximately 28° (21). Lead and trail knee flexion angles were also reduced, measuring 3.0° (4.1°) and 2.5° (3.4°), respectively, compared with normative ranges of 20–25° (22). Other alignment variables were outside the normative ranges, as reported in Table 1.

### Downswing parameters

All downswing angular velocities were substantially below normative ranges (22–24). Maximum pelvis angular velocity reached 91.5°/s (13.9°/s), torso angular velocity was 30.5°/s (6.9°/s), and lead arm angular velocity peaked at 26.3°/s (17.9°/s). Maximum club speed at impact was 21.5 m/s (1.9 m/s), below expected ranges of 36–52 m/s (24, 25). The lead wrist angle at impact remained near neutral, consistent with the monolimb prosthesis design.

### X-factor

Both the pelvis and thorax X-factor amplitudes were reduced compared with normative values (26). Pelvis rotation reached −26.7° (3.8°) and thorax rotation reached −27.2° (4.0°).

### Weight shift

Weight shift patterns at address, transition, and impact fell within normative ranges; however, during follow-through, the participant retained 58.5% (5.9%) of body weight on the trail limb, instead of shifting predominantly to the lead limb (26).

## Discussion

This study aimed to design, fabricate, and evaluate a golf-specific prosthesis for a veteran with bilateral upper limb loss, with a primary focus on feasibility and functional participation. The results demonstrated that the prosthesis enabled independent club attachment, a coordinated two-armed swing, and successful engagement in adaptive golf, addressing an unmet need in a population that faces substantial barriers to sport participation. Biomechanical analysis revealed reduced angular velocities, lower clubhead speed, limited X-factor rotation, and neutral wrist motion relative to normative values (26); however, these findings were intended to characterize movement strategies and identify design-relevant constraints rather than to evaluate athletic performance. Together, the results illustrate how a clinically feasible, user-centered prosthetic design can enable participation in complex sport activities while informing future biomechanical refinement.

Observed biomechanical deficits likely reflected a combination of prosthetic design limitations, functional consequences of bilateral upper limb loss, and the participant's recreational skill level. Published normative biomechanical datasets were used as contextual reference points to characterize the magnitude and nature of these deviations rather than as performance standards. Consistent with prior adaptive sports biomechanics research, deviations from normative values are expected in recreational prosthesis users and reflect interactions among device constraints, user skill level, and individual characteristics; therefore, biomechanical comparisons should be interpreted in the context of functional participation rather than elite performance benchmarks (27). Because bilateral upper limb loss precludes use of an intact limb control, external normative data provided the most appropriate available comparison framework. Accordingly, these findings should be interpreted as descriptive indicators of functional constraints and design opportunities rather than measures of athletic proficiency.

The feasibility of use for this custom prosthesis is clinically meaningful, as participation in adaptive sports has well-established physical and psychosocial benefits, including improved balance, coordination, cardiovascular activity, social connectedness, and quality of life (28–30). For individuals with upper limb loss, opportunities to engage in recreational activities can be restricted due to limitations in prosthesis function and the need for assistive strategies (31). By enabling this participant to golf, the custom prosthesis supported both functional performance and participation goals, aligning with rehabilitation priorities that emphasize health, well-being, and community reintegration (32). Notably, the prosthesis was used in real-world golf settings as part of an adaptive sports clinic (20), including the driving range, chipping and putting greens, and a par-3 executive course, illustrating its practicality and durability outside of the laboratory environment. Beyond participation outcomes, the study also provides insight into biomechanical considerations relevant to sport-specific prosthetic design (15).

Compared with existing golf-specific devices such as the Eagle and Eagle Flex, which provide fixed terminal attachments for club stabilization, the monolimb clamshell design represents a more integrated and individualized structural approach. While the Eagle Flex offers some wrist motion via a flexible interface, its segmented architecture introduces additional interfaces between the socket, device, and club, which may influence multi-plane rotation and energy transfer during the swing (33). Additionally, commercially available devices do not typically accommodate bilateral upper limb loss, limiting their relevance as biomechanical comparators in this case. In contrast, the monolimb design provides a continuous structural frame from socket to distal interface, directly integrating the club into the prosthesis to support user-controlled rotation and secure club coupling. The hinged clamshell mechanism securely encloses the club grip while allowing rapid interchangeability. Importantly, the fabrication techniques employed are clinically feasible, using materials, suspension systems, and socket designs familiar to practicing prosthetists, supporting potential scalability and translation to other patients. To our knowledge, this represents the first reported prosthetic solution facilitating a two-armed golf swing for an individual with bilateral upper limb loss without requiring club modifications or segmented distal attachments.

Biomechanical findings illustrated some of the impact of prosthetic design constraints on swing performance. The fixed, neutral wrist orientation likely contributed to reduced clubhead speed, diminished X-factor rotation, and neutral wrist angles at impact, reflecting a trade-off between mechanical stability and distal segment power generation. Such limitations are consistent with prior reports on adaptive golf prostheses, where restricted motion compromises power generation and kinematic sequencing (19, 33, 34). Iterative modifications based on patient feedback, such as removing the stabilizing handle and redesigning the contralateral prosthesis to reduce irritation, highlight the importance of ongoing co-design between clinicians and athletes to optimize both comfort and function (15).

Beyond prosthetic design, a golf-specific rehabilitation program may help further enhance outcomes. Targeted training to improve postural alignment, X-factor rotation, and weight shift, particularly during follow-through, could correct some of the deviations observed in this study and improve swing efficiency (20, 33). The participant demonstrated rapid off-loading of the lead limb during the follow-through compared with normative values. These observations are descriptive and should not be interpreted as causal effects of the prosthesis alone. Rather, they likely reflect a combination of factors, including conservative balance strategies, prosthesis confidence, and lower limb coordination, as well as individual characteristics such as age and skill level. Improved follow-through mechanics may support more effective transfer of momentum, potentially increasing clubhead speed and overall swing performance. Rehabilitation programs that integrate both prosthetic training and golf-specific skill development may allow users to better adapt to device constraints, improve biomechanical efficiency, and reduce the risk of secondary musculoskeletal discomfort (35). Future studies should consider pairing prosthetic design innovations with structured rehabilitation protocols to help optimize participation and performance.

The biomechanical data collected also provide insight into future design refinements. Incorporating controlled wrist flexion, as suggested by normative wrist angle data (36, 37), may improve clubface control at impact. Additional innovations, such as slotted distal grooves for rapid club positioning or magnet-based quick-release systems, could further streamline club changes and enhance independence, particularly for individuals with bilateral upper limb loss. Coupling these design advances with structured biomechanical assessment and user-centered feedback may foster greater usability and engagement.

Despite promising findings, some limitations should be acknowledged. This study describes a single custom device tailored to one participant's residual limb anatomy, swing style, and participation goals. As a single-participant study, inferential statistical analyses were not performed, limiting generalizability to other individuals with bilateral upper limb loss. Furthermore, only acute biomechanics were assessed in a controlled laboratory setting, and although the prosthesis was used successfully in real-world golf activities, longer-term performance and measures of patient-reported satisfaction, comfort, durability, and participation outcomes were not systematically collected in these settings. Additionally, comparisons to normative biomechanical data should be interpreted with caution. As a single-case study, this work was not designed to establish causal relationships or to directly benchmark performance against professional golfers. Normative data serve as a contextual reference to illustrate potential deviations in movement patterns and should not be used to infer efficacy or superiority of the prosthetic design.

Future work should extend beyond mechanical refinement to include objective monitoring of device use and outcomes. Incorporating wearable sensors, such as inertial measurement units, would enable field-based assessment of biomechanics during real-world play (38, 39). Combining these data with patient-reported outcomes, such as the Patient-Reported Outcomes Measurement Information System Satisfaction with Participation in Discretionary Social Activities survey (40, 41), would provide deeper insight into functional performance, durability, and psychosocial impact. Expanding this research to a small cohort of golfers with bilateral upper limb loss could help establish biomechanical benchmarks, identify inter-individual variability, and guide both iterative device development and rehabilitation strategies to advance access to adaptive sports for individuals with bilateral upper limb loss.

## Conclusion

This study demonstrated the feasibility of a customized, golf-specific prosthesis that enabled meaningful participation in adaptive sports for an individual with bilateral upper limb loss. The device allowed the participant to securely swing the golf club, independently change clubs, and engage in golf, representing a significant achievement for a population that faces substantial barriers to recreational activity. Although biomechanical analysis revealed deviations relative to able-bodied golfers, such as reduced angular velocities, diminished X-factor, and limited wrist motion, these findings are descriptive and likely reflect a combination of prosthetic constraints, participant skill, and individual characteristics, highlighting areas for potential design improvement. Importantly, the prosthesis was fabricated using techniques and materials readily available to practicing prosthetists, supporting potential broader translation. Overall, this study emphasizes the importance of individualized, user-centered prosthetic solutions that prioritize both functional performance and participation, advancing access to adaptive sports for individuals with bilateral upper limb loss.

## Data Availability

The original contributions presented in the study are included in the article/Supplementary Material, further inquiries can be directed to the corresponding author.

## Supplementary material

Supplementary Table S1 Parameter definitions, calculations, and orientations.
