# Supplementary material for: Novel golf prosthesis for bilateral upper limb loss: design, fabrication, and biomechanical evaluation
Source: Front Sports Act Living. 2026 May 8;8:1766695. doi: 10.3389/fspor.2026.1766695 (PMC13194480; doi:10.3389/fspor.2026.1766695)
Supplement: Supplementary Table S1 — Parameter definitions, calculations, and orientations. [file Table1.docx]

**Supplementary Table S1: Parameter definitions, calculations, and orientations**

| **Body Position at Address** | | |
| --- | --- | --- |
| *Parameter* | *Definition/Calculation* | *Orientation* |
| Stance width (cm) | Distance between the centroids of the lead and trail foot | - |
| Stance percentage of the shoulders (%) | Percentage comparison of the stance width, compared with the distance between the left and right acromion markers. | >100% = wider than shoulder width; <100% = narrower than shoulder width |
| Spine forward tilt (^o^) | Angle between the major axis of the pelvis and the thorax in the sagittal plane | Positive for forward tilt, 0^0^ for neutral position |
| Spine side tilt (^o^) | Angle between the major axis of the pelvis and the thorax in the frontal plane | Positive for bending  towards the trail leg, 0^0^ for neutral position. |
| Knee flexion (^o^) | Angle between the major axis of the shank and thigh segments | Positive for flexion, 0^0^ for neutral position. |
| **Downswing Parameters** | | |
| Maximum Pelvis Angular Velocity (^o^/s) | Angular velocity of the pelvis, calculated relative to the major axis of the pelvis | Positive for rotating  forward |
| Maximum Torso Angular Velocity (^0^/s) | Angular velocity of the torso, calculated relative to the major axis of the thorax | Positive for rotating  forward |
| Maximum Lead Arm Angular Velocity (^o^/s) | Angular velocity of the lead arm, calculated relative to the vertical axis of the lab coordinate system | Positive for rotating  forward |
| Maximum Club Angular Velocity (^o^/s) | Angular velocity of the club, calculated relative to the vertical axis of the lab coordinate system | Positive for rotating  forward |
| Lead Wrist Angle at Impact (^o^) | Angle between the major axis of the hand and lower arm | Positive for curled wrist,  0^o^ for neutral |
| Maximum Club Speed (m/s) | Measured as the maximum linear velocity of the distal club shaft marker |  |
| **X-factor** | | |
| Pelvis (^o^) | Pelvis rotation angle is the angle of the pelvis in the axial plane, defined by the orientation of the line through the right and left anterior superior iliac spines relative to the laboratory coordinate system. X-factor is the angle between the line through the right and left anterior superior iliac spines and the line through the right and left acromion processes in the axial plane, representing the rotational separation between the pelvis and thorax segments. | Negative when upper pelvis leads the torso |
| Thorax (^o^) | Thorax rotation angle is the angle of the thorax in the axial plane, defined by orientation of the line through the right and left acromion processes relative to the laboratory coordinate system. X-factor is the angle between the line through the right and left anterior superior iliac spines and the line through the right and left acromion processes in the axial plane, representing the rotational separation between the pelvis and thorax segments. | Negative when upper pelvis leads the torso |
| **Weight Shift** | | |
| Weight shift between lead and trail leg (%) | Percentage of vertical ground reaction force ratio between the lead and trail legs. | - |
